# Micafungin versus Amphotericin B in treatment of invasive fungal infection in preterm neonates: a randomized control trial

**DOI:** 10.1186/s13052-025-01852-9

**Published:** 2025-02-27

**Authors:** Mariam John Amin Ibrahim, Marwa Saad Mohammed Fathy, Mertte Ashraf Thabet Ghobrial, Maha Hassan Mohamed

**Affiliations:** 1 Pediatrics and Neonatology, Faculty of Medicine, Ain Shams University, Cairo, Egypt; 2 Egyptian Ministry of Health and Population, Masr El Hora Hospital, Minia, Egypt; 3 Professor of Medical Microbiology and Immunology, Faculty of Medicine, Ain Shams University, Cairo, Egypt

**Keywords:** Invasive fungal infection, Micafungin, Amphotericin B, Preterm, Neonates, Candida non albicans

## Abstract

**Background:**

Micafungin, Amphotericin B, and Fluconazole are the primary therapeutic agents employed to address invasive fungal candidiasis in neonates. Resistance to fluconazole is gradually developing in neonatal intensive care units. We aimed to conduct a comparative analysis of Micafungin and Amphotericin B in terms of their effectiveness and safety in the treatment of invasive fungal infections in neonates.

**Methods:**

Fifty-six preterm neonates with invasive fungal infection proven by fungal culture and who had received fluconazole for at least one week were included in our study and were divided randomly into two groups. Micafungin group: twenty-eight preterms received Micafungin at a dose of 8 mg/kg/day for 14 days. Amphotericin B group: twenty-eight preterms received amphotericin B at a dose of 1 mg /kg/day for 14 days. Clinical and laboratory follow up by fungal culture were performed after 14 days.

**Results:**

Neonates in the Micafungin group showed significant increased percentage for complete cure of the fungal infection compared to Amphotericin B group 18(64.3%) vs. 10(35.7%) respectively and decreased percentage of incomplete cure 10(35.7%) vs. 18(64.3%) respectively with *p*-value 0.030. A higher percentage of neonates were completely cured for both candida albicans (65.2%) and non-albicans (60%) in the micafungin group. Duration of respiratory and circulatory support was significantly shorter also. No additional drug side effects were observed with Micafungin except for mild hypomagnesemia. There was an increase in blood urea nitrogen with Amphotericin B.

**Conclusion:**

Micafungin is effective and well tolerated for the treatment of invasive fungal infections in preterm neonates.

**Trial registration:**

The current study was approved by clinicaltrials.org and the protocol ID NCT06413056 was retrospectively registered in on 11th of march 2024. https://clinicaltrials.gov/study/NCT06413056?cond=micafungin%20in%20neonates&rank=2.

## Background

The incidence of invasive fungal infection (IFI) in neonates has increased over the past two decades, as survival has increased among preterm neonates. In the neonatal intensive care unit (NICU), the predominant invasive fungal infections caused by Candida species are caused by *Candida albicans* and *Candida parasilosis* [1].

Micafungin, amphotericin B deoxycholate, and fluconazole are recommended by the European Society for Clinical Microbiology and Infectious Diseases (ESCMID) as first line treatments of invasive candidiasis in neonates [1].

Micafungin is one of three echinocandins presently accessible that serve as primary therapeutic alternatives for candidiasis and candidaemia. It is prescribed for the prophylaxis of Candida infections, which are commonly observed in immunocompromised patients, as well as for the treatment of invasive candidiasis [2].

Usage of high dose of micafungin (8 to 15 mg/kg/day) for infants and neonates with invasive candidiasis is recommended now [3].

In this study we explored the effectiveness and safety of micafungin for the treatment of candidiasis after fluconazole for at least one week for preterm neonates with invasive fungal infection and compared it to Amphotericin B treatment.

## Methods

### Subjects

We conducted this prospective randomized controlled clinical trial on neonates less than 36-week gestational age within the intensive care unit for newborns (NICU) children’s hospital, Ain-Shams University hospitals, Cairo, Egypt, from September 2022 till September 2023. All the patients had signs and symptoms of invasive fungal infection which included fever, tachypnea, tachycardia, respiratory distress, pulmonary consolidation, increased oxygen requirements and assisted ventilation either invasive or noninvasive, apnea or bradycardia [4]. Invasive fungal infection was proven by fungal culture and these patients had already received fluconazole for at least one week.

Any neonate with hepatic dysfunction for any cause (hepatitis or hepatic failure), or with elevation in aspartate aminotransferase (AST), alanine transaminase (ALT), alkaline phosphatase, hypertension, neutropenia, or thrombocytopenia, with elevated renal function or with arrythmia was excluded.

### Methods

The 56 preterm neonates who received fluconazole for at least one week were randomly allocated to either one of the two groups; Micafungin group: twenty-eight (28) preterm neonates who received a 14-day course of 8 mg/kg/day of micafungin [5] and Amphotericin B group: twenty-eight (28) preterm neonates received amphotericin B at a dose of 1 mg /kg/day for 14 days [6].

Fungal culture was done before the start of the treatment to confirm the presence of invasive fungal infection and after 14 days to assess the effectiveness of the drug. A complete cure was defined as no growth in the fungal culture after 14 days of the designated drug, an incomplete cure was defined as the absence of the original organism but the growth of another fungal species after 14 days, and failure of treatment was the persistence of the initial organism after 14 days.

CBC, CRP, Na, K, Ca, Mg, blood urea nitrogen, and creatinine were assessed initially, after 7 days and after 14 days of completing the treatment.

A Complete assessment was done during hospital admission for all the following: detailed antenatal history if the mother had any antenatal diseases. Natal history: Mode of delivery either normal vaginal delivery or cesarean section, prolonged labor and cord prolapse, amniotic fluid (normal, offensive, meconium stained), resuscitation by positive pressure ventilation, endotracheal intubation, oxygen administration, or chest compressions, medications given, Apgar score at 1, 5 and 10 min. Postnatal history: Determination of gestational age. Cause of NICU admission. Full clinical examination included chest auscultation three times daily to assess air entry, crepitation, and wheezes, cardiac pre and post ductal saturation assessment and auscultation for cardiac murmur, abdominal palpation for organomegaly or tenderness, vomiting, diarrhea and feeding intolerance. Neurological examination: measurements for occipitofrontal circumference, assessment of anterior and posterior fontanelles, assessment of tone and reflexes. The end point of the study was after completing 14 days of treatment with the designated drugs, or if the patient showed significant complications after the use of the drug. Monitoring of side effects of micafungin as nausea, vomiting, diarrhea, rash, arrhythmia, hypertension, hepatic dysfunction, hepatitis, elevation of liver enzymes, hypomagnesemia, neutropenia, thrombocytopenia [7]. Monitoring side effects of amphotericin B as anemia, nausea, vomiting, fever, chills, thrombocytopenia, hypokalemia, and impaired renal function [8].

### Consent and ethical consideration

This study was done after approval from the Research Ethics Committee, Ain Shams University (number: M S 654/2022). Informed written consent was taken from the parents or the legal guardians of the eligible neonates after explanation of the study and its aim. The included participants had the right to withdraw from the study at any time without giving any reasons. Data was collected confidentially and was only used for research purposes.

### Statistical analysis

The Package pertaining to the social sciences, version 23.0 (SPSS Inc., Chicago, Illinois, USA) was used for statistical analysis. When the distribution of the quantitative data was parametric, it was expressed as the mean ± standard deviation and range. Conversely, for non-parametric data, which followed a non-normal distribution, the median was utilized in conjunction with the inter-quartile range (IQR). Furthermore, quantitative data and percentages were used to represent qualitative variables. The normality of the data was examined utilizing the Kolmogorov-Smirnov and Shapiro-Wilk tests. The t-test of significance was employed to compare the means of two groups, while the Mann Whitney U test was utilized for non-parametric data. To compare qualitative data across groups, the chi-square test was applied. The accepted margin of error was 5%, and the confidence interval was established at 95%. The *p*-value was deemed significant in the subsequent manner: Probability (*P*-value) values below 0.05 were deemed significant, while values below 0.01 were deemed highly significant.

## Results

**Fig. 1:**
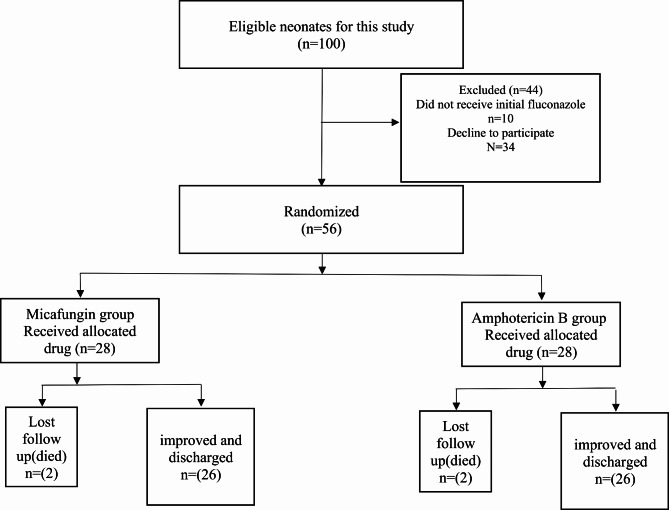
Consort of the studied neonates

Out of the 100 patients who were eligible to participate in the study, 44 were excluded due to either failure to complete the course of fluconazole or refusal to participate. Fifty-six (56) patients were later randomized to either of the 2 groups, 2 in each group either lost follow up or died, as shown in Fig. 1.

The clinical demographic data and clinical diagnoses of the studied neonates are shown in Table 1 and were comparable between both groups. Micafungin group showed a significant higher percentage 64.3% for completely cured patients and a significantly lower percentage 35.7% of incomplete cured patients compared to Amphotericin B (35.7%, 64.3% respectively). A significant greater percentage of patients were completely cured with the use of micafungin. On the other hand, the use of amphotericin B failed to attain a high percentage of complete cure, and most of the patients were partially treated (64.3%) Table 2.

**Table 1:** Demographic characteristics, mode of delivery and diagnosis of the studied neonates in both groups

		Micafungin *N* = 28	Amphotericin B *N* = 28	*P* value	Sig.
		Mean ± (SD)	Mean ± (SD)		
Gestational age (weeks)		34 ± 1.5	33.6 ± 1.9	0.606	NS
Weight (gm)		**1885.7 ± 371.6**	**2197.1 ± 591.7**	**0.032**	**S**
Length (cm)		42.3 ± 3.5	44.4 ± 3.5	0.09	NS
Head circumference (cm)		30.7 ± 1.8	31.5 ± 2.4	0.189	NS
Patient age at admission (days)		9 ± 9.7	10.4 ± 9.8	0.182	NS
Patient age at onset of fungal infection (days)		29.2 ± 11.2	32.7 ± 24.7	0.974	NS
		**N. (%)**	**N. (%)**		
Mode of delivery	CS	22 (78.6%)	20 (71.4%)	0.379	NS
	NVD	6 (21.4%)	8 (21.4%)		
Diagnosis	Surgical	4 (8.3%)	4 (8.2%)	1	NS
	RD	10 (20.9%)	11 (22.4%)	0.553	NS
	Congenital heart disease	4 (8.3%)	4 (8.2%)	1	NS
	Congenital anomalies	8 (16.7%)	12 (24.5%)	0.15	NS
	sepsis	22 (78.6%)	18 (64.3%)	0.237	NS

Mann Whitney U test. Chi square test. SD: standard deviation, CS: cesarean section, RD: respiratory distress, NVD: normal vaginal delivery, n: number, (%):percentage. Sig.: significance NS: not significant, (*P*-value > 0.05), S: significant (*P*-value < 0.05 ), HS: highly significant (*P*-value < 0.01)

**Table 2:** Comparison between the frequency of complete cure in both studied groups

	Micafungin *N* = 28		Amphotericin B *N* = 28		Test value	*P*-value	Odds Ratio	Sig.
	Cured no.	%	Cured no.	%				
Complete cure	18	64.3	10	35.7	0.900	0.030	3.240	S
Incomplete cure	10	35.7	18	64.3	0.438			

Fisher’s exact test, no: number, %: percentage, Sig.: significance S: significant (*P*-value < 0.05)

**Fig. 2:**
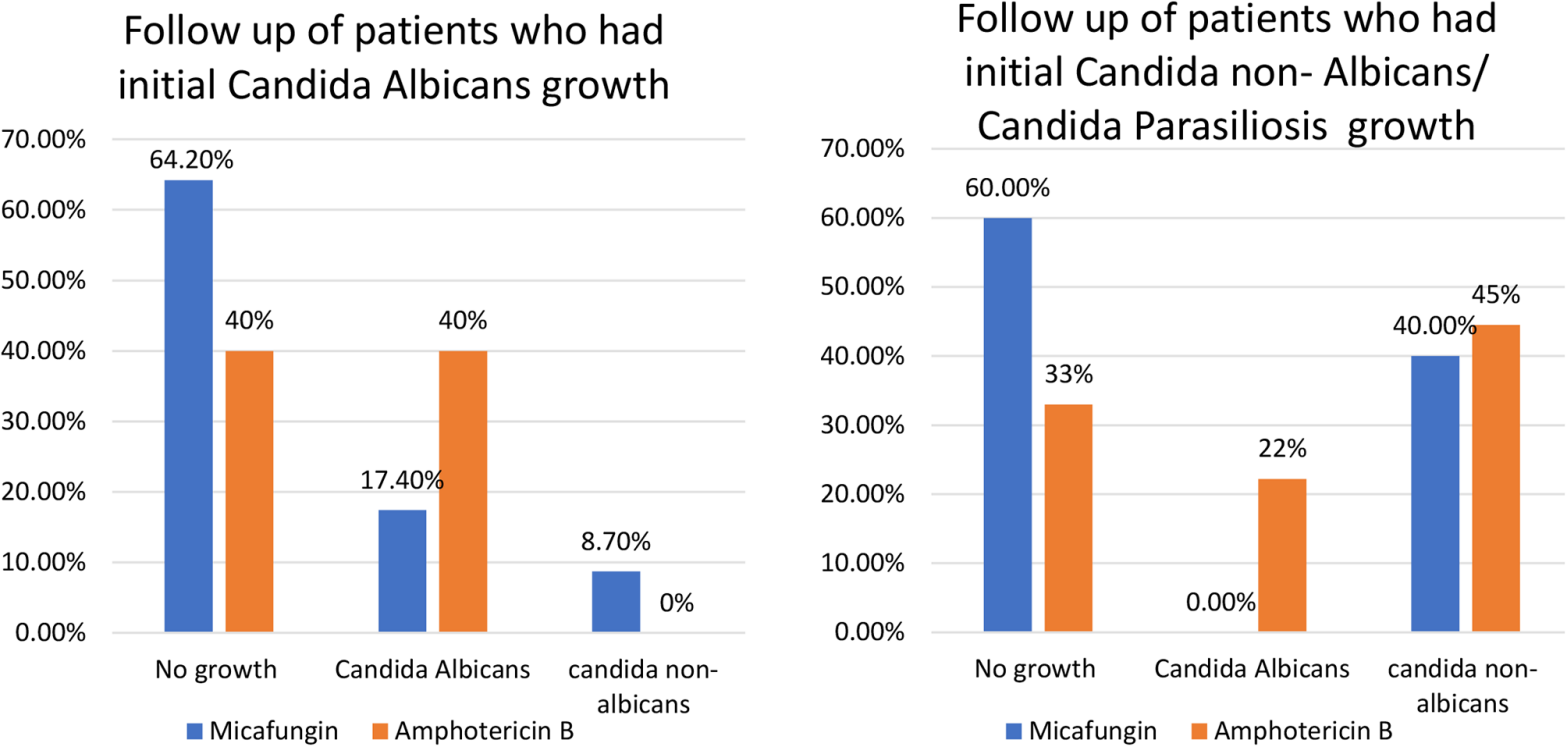
Frequency of the cured patients according to the type of fungal species in both studied groups

The comparison of the frequency of cured patients according to type of fungal species in both studied groups is shown in Table 3; Fig. 2. In micafungin group: 23 patients had initial growth of *candida albicans*; 65.2% were completely cured, 17.4% had persistent *candida albicans* in fungal culture and 8.7% were cured of the *candida albicans* but developed an infection with *candida non- albicans (candida parasiliosis)*. Five (5) patients of the same group had initial *candida non albicans* growth *(candida parasiliosis)*, 60% were completely cured and 40% had persistence of the fungal species in the fungal culture.

**Table 3:** Comparison of the frequency of different fungal species in follow up fungal cultures among the studied neonates

			Micafungin			Amphotericin B		Test value	*P*-value	Sig.
			*N* = 28			*N* = 28				
Fungal culture on admission	Fungal culture on follow-up		**no.**	**(%)**		**no.**	**(%)**			
Candida albicans	No growth	23	15	65.2	10	4	40	2.513	0.113	NS
	Candida albicans		4	17.4		4	40	1.489	0.222	NS
	Candida non albicans/ candida parasiliosis		2	8.7		0	0	1.016	0.313	NS
	Death		2	8.7		2	20	0.645	0.422	NS
Candida non albicans*/ candida parasiliosis*	No growth	5	3	60	18	6	33.3	1.168	0.28	NS
	Candida albicans		0	0		4	22.2	1.345	0.246	NS
	Candida non albicans/ candida parasiliosis		2	40		8	44.5	0.031	0.859	NS
Total			28	100		28	100			

No: number, (%): percentage Chi- square test, Sig.: significance NS: not significant, (*P*-value > 0.05)

In amphotericin B group, 10 patients had initial growth of *candida albicans*; 40% were completely cured, 40% had persistent *candida albicans* in fungal culture. 18 patients of the same group had initial *candida non albicans (candida parasiliosis)* growth, 33.3% were completely cured and 44.5% had persistence of the fungal species in the fungal culture, 22.2% were cured of the *candida non- albicans (candida parasiliosis)* but developed an infection with *candida albicans.*

The comparison of the outcome of the treatment between micafungin and amphotericin groups according to the species of the fungal organisms cultured was non-significant as seen in Table 3.

Neonates in the Micafungin group showed significantly shorter duration of respiratory support and circulatory support than neonates in the Amphotericin B group as seen in Table 4.

**Table 4:** Comparison of management between the two studied groups

		Groups		*P* value	Sig.
		Micafungin *N* = 28	Amphotericin B *N* = 28		
Respiratory support	CPAP	4(14.3%)	CPAP 2(7.1%)	0.194	**NS**
	CPAP and NIPPV	0(0.0%)	2(7.1%)	0.154	**NS**
	MV	8(28.6%)	11(39.3%)	0.402	**NS**
	NIPPV	0(0.0%)	2(7.1%)	0.154	**NS**
	No respiratory support	16(57.1%)	11(39.3%)	0.186	**NS**
Duration of respiratory support (days)		13.5 ± 6.1 5–25	21.5 ± 11.1 2–40	0.008	**HS**
Circulatory support	1st line	1(3.6%)	1(3.6%)	1.000	**NS**
	2nd line	27(96.4%)	27(96.4%)	1.000	**NS**
Duration of circulatory support (days)		9.1 ± 3.4 5–14	12.5 ± 1.8 10–14	0.024	** S**

Fisher’s exact test, Mann Whitney U test. First line: Dopamine, dobutamine, second line: adrenaline, noradrenaline

CPAP: continuous positive airway pressure NIPPV: non-invasive positive pressure ventilation MV: mechanical ventilation (%): percent. Sig.: significance NS: not significant, (*P*-value > 0.05), S: significant (*P*-value < 0.05 ), HS: highly significant (*P*-value < 0.01)

Outcome of the studied neonates after antifungal treatment is shown in Table 5. Although age at discharge and the length of hospital stay were both less in the micafungin group than the Amphotericin group however it did not mount to statistical significance.

**Table 5:** Outcomes of the studied neonates after antifungal treatment

				*P* value	Sig.
		Micafungin *N* = 28	Amphotericin B *N* = 28		
Patient age on	Mean ± SD	58.6 ± 19.5	72.3 ± 29	0.497	NS
discharge (days)					
Length of stay (days)	Median (IQR)	50 (60 − 40)	60 (66.0–40)	0.182	NS
Died	**N. (%)**	**2 (7.1%)**	**2 (7.1%)**	**1**	**NS**
Discharged		26 (92.9%)	26 (92.9%)		
Complications		0	0		

Fisher’s exact test & Mann Whitney U test N: number, (%): percentage, SD: standard deviation, IQR: interquartile range, Sig.: significance NS: not significant, (*P*-value > 0.05)

**Fig. 3:**
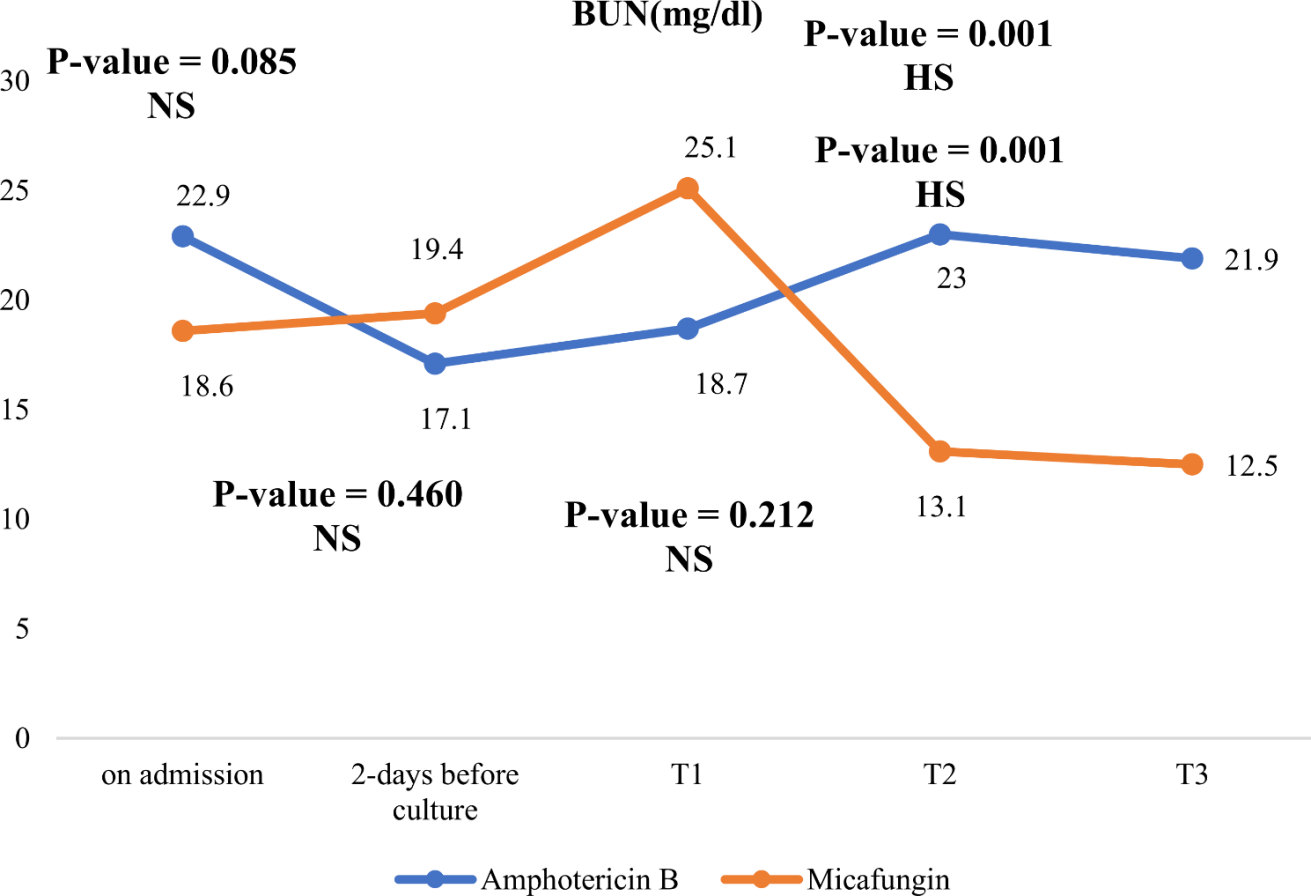
Changes in BUN levels during NICU stay in both studied groups. NS: not significant, (*P*-value > 0.05), HS: highly significant (*P*-value < 0.01)

**Fig. 4:**
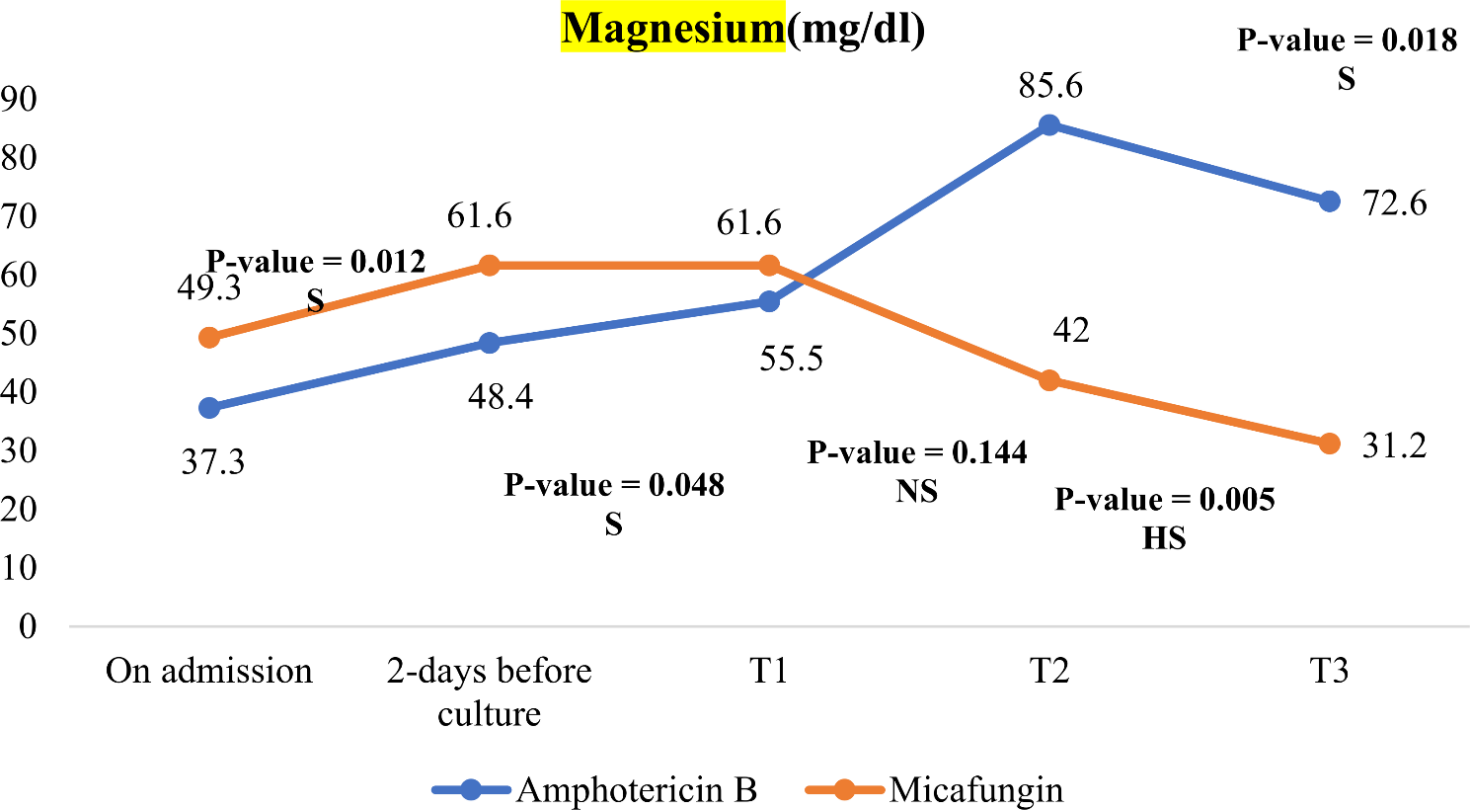
Changes in magnesium level during NICU stay in both studied groups. NS: not significant, (*P*-value > 0.05), S: significant (*P*-value < 0.05), HS: highly significant (*P*-value < 0.01)

**Fig. 5:**
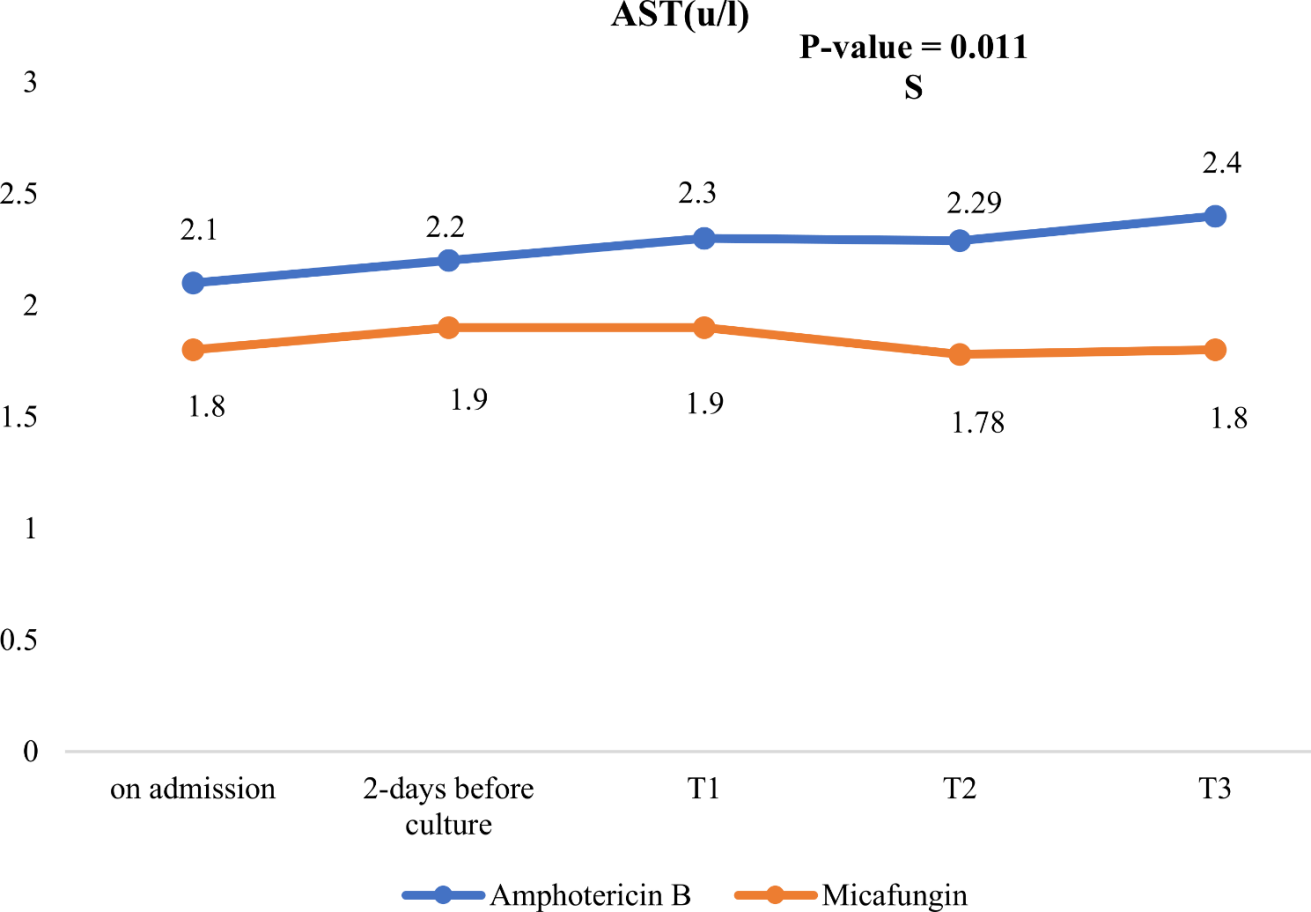
AST level changes during NICU stay in both studied groups. S: significant (*P*-value < 0.05)

In the follow up after 14 days of antifungal treatment, micafungin group showed significantly lower BUN, lower magnesium level and lower ALT levels compared to Amphotericin B group as evident in Table 6; Figs. 3, 4 and 5.

**Table 6:** Laboratory data of the studied neonates after 14 days of antifungal treatment

Laboratory data after14 days of treatment	Groups		*P* value	Sig.
	Micafungin	Amphotericin B		
	*N* = 28	*N* = 28		
	Mean ± SD	Mean ± SD		
Bun(mg/dl)	**12.5 ± 9.7**	**21.9 ± 13**	**0.001**	**HS**
creatinine(mg/dl)	0.3 ± 0.2	0.3 ± 0.1	0.699	NS
Na(mg/dl)	135.6 ± 5.6	138.7 ± 7.2	0.1	NS
K(mg/dl)	4.4 ± 0.9	4.5 ± 0.7	0.335	NS
Calcium(mg/dl)	9.6 ± 0.9	8.9 ± 1.8	0.232	NS
Magnesium(mg/dl)	**1.8 ± 0.3**	**2.4 ± 1**	**0.018**	**S**
ALT(u/l)	**31.2 ± 20.1**	**72.6 ± 63.2**	**0.011**	**S**

Mann Whitney U test. BUN: blood urea nitrogen, Na: sodium, K: potassium, ALT: alanine transaminase, N: number of patients, Sig.: significance NS: not significant, (*P*-value > 0.05), HS: highly significant (*P*-value < 0.01)

## Discussion

Proper management of fungal infections in neonates especially preterms is crucial for better outcome in NICUs worldwide. The widespread use of antibiotics has led to the flourishing of fungal species in preterm neonates. Researching the effectiveness of the different antifungal drugs proposed was the target of our study, and we studied the effectiveness of micafungin in comparison to amphotericin B in the management of invasive fungal infections in preterm neonates. In our study Micafungin group showed significant increased percentage of complete cure of neonates with fungal culture changing to negative after fourteen days of treatment, compared to Amphotericin B group, 18 (64.3%) vs. 10 (35.7%) respectively and a decreased percentage of incomplete cure compared to Amphotericin B, 10(35.7%) vs. 18(64.3%) respectively with *p*-value 0.030. Amphotericin B was not able to attain complete cure after 14 days of treatment with a persistent growth of fungal species in fungal culture after 14 days of treatment.

Similar results were reported in a previous study [1] in which the effectiveness of Micafungin in altering negative fungal culture was attained at 5.5 days ranging (1-11days). The duration was extended in *candida glabrata*, meningitis and in patients with candida albicans urinary sepsis to 9 days.

Previous prospective, observational studies, have reported that patients under the age of 18 with invasive candidiasis have achieved favorable treatment outcomes and high rates of survival with micafungin [9, 10]. The success rates observed in neonates are comparable to those determined in pediatric research. On the other hand, an illustration provided by the researchers documented comparable rates of treatment efficacy after treatment with Micafungin at a dose of 2 mg/kg, and liposomal amphotericin B 3 mg/kg in patients < 16 years of age with invasive candidiasis [11].

According to the results we reached in our study we recommend completion of fungal treatment with Micafungin for at least 14 days to attain fungal eradication in low-resource countries where fungal culture will not be readily available to be requested as frequently as needed.

In our study, regarding the different candida species in the Micafungin group, 65.2% of the patients who had original *candida albicans* growth in fungal culture, had total eradication of this organism in the blood culture after 14 days of taking the treatment, on the other hand only 40% of the patients who received Amphotericin B had complete eradication from *candida albicans* in their fungal culture.

Only 17.4% of the patients that had original *candida albicans* growth in their fungal culture, showed persistence of the organism after 14 days of treatment with Micafungin, as opposed to a greater percentage (40%) of the patients that similarly showed persistence of the *candida albicans* species in Amphotericin B group.

A total of 8.7% of the patients who had original *candida albicans* growth in their fungal culture and received Micafungin for 14 days, developed *candida non albicans* in their fungal blood culture after 14 days. We could not estimate the exact time when the patient developed the non albicans species infection, so effectiveness of the drug cannot be assessed according to this issue on its own.

60% of the patients with original *candida non-albicans* growth in their fungal culture and received Micafungin had total eradication of the organism after 14 days of taking the treatment, on the other hand a lower percentage of 33.3% of the patients who received Amphotericin B had total eradication from *candida non-albicans* in their fungal culture.

40% (40%) of patients with original *candida non-albicans* growth in their fungal culture and who received Micafungin for 14 days still exhibited growth of *candida non-albicans* in their fungal culture, which is similar to 44.5% of patients who received Amphotericin B and still had persistence of *Candida non-albicans* in their fungal culture after 14 days of treatment. The previous comparisons showed a clear difference in percentages but did not mount statistical significance owing to the small sample size used. Further studies are recommended to be done to further tackle the eradication of specific fungal species in each category of neonates.

Similarly, ***Benjamin et al.*** [12] reported in his study, that was done on 20 infants who received Micafungin and 10 infants who received Amphotericin B deoxycholate for at least 21 days and fungal culture was done one week after the last dose, that complete eradication time was observed in 11 (55%) of patients who received Micafungin and 8(80%) who received Amphotericin B deoxycholate. Candida infections that persisted were due to *Candida parapsilosis* in 2 Micafungin-treated infants and *Candida glabrata* and *Candida albicans* in neonates treated with amphoteric acid deoxycholate (*n* = 1 for each). In fact, one-fourth of invasive fungal infection cases in very low birthweight neonates (less than 1500 g) are attributable to parapsilosis [14]. Among non-albicans Candida species, parapsilosis has become the most prevalent infection in neonatal invasive candidiasis [15].

In our study, we used a high dose of micafungin in neonates due to high clearance [13] and high volume of distribution in neonates as presented by previous studies [3].

Notably, there is an uncertainty about the clinical applicability of echinocandins in the treatment of *Candida parapsilosis* as it needs a mean inhibitory concentration that is greater in comparison to other Candida species [16].

In our study, the Micafungin group did not show any abnormalities in liver or kidney function; however they had significantly lower magnesium concentration compared to Amphotericin B. The drop in magnesium showed no signs or symptoms and did not need any correction. Creatinine and ALT concentrations were within normal acceptable range indicating no affection or alteration with the use of the Micafungin. On the other hand, Amphotericin B patients had significant elevation of their renal function compared to Micafungin group.

In agreement with our results ***Arıkan et al.*** [1] also reported no increase in liver function test or renal function test on using Micafungin. Conversely, a previous study reported elevation in liver, renal functions, and electrolytes in Micafungin group. In a study by ***Benjamin et al.*** [12] the authors reported significant elevations in bilirubin and liver enzymes in the Micafungin group than Amphotericin B group. In addition, there was a significant drop in potassium and magnesium levels which needed to be treated by giving the respective deficit of potassium and magnesium. This study was in infants from 2 to 120 days, who received Micafungin at 10 mg/kg/day for a minimum of 21 days and a maximum of 28–42 days. The basal liver function of the patients was not known which may explain the elevation of liver function after the use of micafungin.

In our study subjects who received Micafungin did not develop any complications, indicating the safety of the use of Micafungin with no emergence of complications.

In our study as part of the improvement of the patient’s general condition patients in Micafungin group experienced a significant decrease of duration of respiratory support and were successfully extubated earlier than the patients in the Amphotericin B group. Additionally, the positive effect on circulation was observed in the readiness to withdraw inotropic drugs and the short duration of total inotropes used.

Although subjects in micafungin group in our study showed short duration of respiratory and circulatory support, this was not interpreted in the length of hospital stay or the age at discharge. It should be noted that the patients included in the study had other comorbid conditions that could have prolonged their hospital stay until everything was completely managed and treated.

To the best of our knowledge hospital stay and age at discharge were not researched in other studies investigating the effect of Micafungin in neonates.

Many preventive strategies have been proposed to minimize fungal, bacterial and viral infections. The most important is the tight adherence to infection control policies implemented in the hospital with designated personnel for this issue, as well as disinfection, environmental cleaning and central line bundles [17].

Several proposed strategies have been introduced to minimize viral infections in NICUs as using the Rota virus vaccine which is a very dangerous infection to this vulnerable age group, especially preterm neonates. Beside the severe dehydration, Rota virus has been reported to cause severe acute pancreatities in children. The research done showed promising results and effective vaccination of neonates in NICUs using a monovalent Rota virus vaccine [18, 19].

Fungal prophylaxis has been proposed especially in preterm neonates using low dose fluconazole. New strategies are now emerging as the use of nystatin, bovine lactoferrin, probiotics, reduced use of H_2_ blockers and broad spectrum antibiotics or prophylaxis with micafungin in special situations, which have all shown favorable results in fungal prophylaxis [20].

The use of antifungal prophylaxis and other methods to reduce fungal infection have been shown to decrease invasive fungal infection in NICUs, but do not prevent it completely. So, clinicians should be at a high level of suspicion to early diagnose and promptly start the effective treatment necessary for this severe condition.

## Conclusions

In conclusion, for the treatment of invasive fungal infections in neonatal intensive care unit patients, micafungin is efficacious and well tolerated. Additionally, Micafungin treatment for neonates with invasive candida resulted in a high success rate and fewer complications than Amphotericin B. Further studies should be performed on a greater scale.

## Data Availability

The data sets generated and analyzed for the study are available from the corresponding author upon reasonable request.

## Abbreviations

IFI: Invasive fungal infection
NICU: Neonatal intensive care unit
ESCMID: European Society for Clinical Microbiology and Infectious Diseases
PROM: Premature Rupture of Membranes
IQR: Inter Quartile Range
SD: Standard deviation
CS: Cesarean section
RD: Respiratory distress
NVD: Normal vaginal delivery
n: Number, (%):percentage
Na: Sodium
K: Potassium
Ca: Calcium
BUN: Blood Urea Nitrogen
CBC: Complete Blood Count
AST: Aspartate Aminotransferase
ALT: Alanine Transaminase
CRP: C-reactive protein
CPAP: Continuous positive airway pressure
NIPPV: Non-invasive positive pressure ventilation
MV: Mechanical ventilation
